# Academic stress is associated with emotional eating behavior among postgraduate students

**DOI:** 10.1186/s12889-026-27233-3

**Published:** 2026-04-01

**Authors:** Mahitab Hanbazaza, Sondos Alsayed, Areej Bawajeeh

**Affiliations:** Department of Food and Nutrition, Faculty of Human Sciences and Design, King Abdulaziz University, Jeddah, 22258 Saudi Arabia

**Keywords:** Academic stress, Emotional eating, Postgraduate students, Saudi Arabia

## Abstract

**Background:**

Academic stress has been linked to unhealthy coping behaviors such as emotional eating. This study examined the association between academic stress and emotional eating behavior among postgraduate students at King Abdulaziz University, Saudi Arabia.

**Methods:**

A cross-sectional study was conducted among a convenience sample of postgraduate students. Data were collected using an online, self-administered questionnaire that included sociodemographic characteristics, Grade Point Average (GPA), Students’ Stress Inventory (SSI), and the Emotional Eater Questionnaire (EEQ).

**Results:**

The sample included 505 postgraduate students (85.9% female). Approximately 50% of participants experienced moderate academic stress, and 46% were emotional eaters. Academic stress was significantly associated with female students (*P* < 0.001), lower income levels (*P* = 0.007), and low levels of physical activity (*P =* 0.007). A statistically significant positive association was observed between academic stress and emotional eating (*P* < 0.001). Students with severe academic stress had significantly higher odds of being more severe emotional eaters (OR = 7.382, 95% CI [3.777, 14.43], *P* < 0.001). This association remained significant after adjusting for demographic, psychological, and lifestyle factors (adjusted OR = 6.633, 95% CI [3.294, 13.343], *P* < 0.001). However, the moderation analysis indicated that sex did not significantly modify the association between academic stress and emotional eating.

**Conclusions:**

These findings highlight the importance of implementing stress management and nutrition interventions to help postgraduate students adopt healthier coping strategies to manage academic stress.

**Supplementary Information:**

The online version contains supplementary material available at 10.1186/s12889-026-27233-3.

## Background

Stress among postgraduate students is a growing concern that has received greater attention due to its significant effects on mental health and well-being. The World Health Organization (WHO) defines stress as a condition characterized by worry or psychological tension caused by challenging or difficult situations [1]. Exposure to prolonged stress has been associated with various health problems, including headaches, sleep disturbances, hypertension, anxiety, depression, and stroke [2]. Among university students, stress may arise from multiple sources, including psychological, emotional, psychosocial, and academic [3]. Academic stress is defined as the anxiety that arises during the educational journey; it has become a widespread issue among students across cultures, countries, and ethnic groups [4, 5]. It is a growing concern among university students, with prevalence rates varying across countries depending on study populations and cultural contexts. For example, the prevalence in Nigeria was around 18% [6], in Mexico 70% [7], in Saudi Arabia 86% [8], and 92% in Sudan [9].

In terms of severity, academic stress can be categorized as mild, moderate, or severe [10], depending on external and internal factors and individuals’ coping abilities [11]. Several factors contribute to academic stress among university students, including study workload, assignments, exams, parental and self-expectations, feelings of hopelessness, and a demanding curriculum [10]. Yet, postgraduate students are particularly vulnerable to academic stress, especially during the thesis-writing stage, which requires constant focus, thorough research, data analysis, and strict adherence to tight deadlines [12, 13]. This period is often associated with an increase in students’ desire for perfection and fear of failure, all of which elevate stress levels [14, 15]. Previous studies have also suggested that academic stress may differ by sex, with some evidence indicating that female students may experience higher levels than males; however, these differences are not consistently observed and may vary across sociocultural and environmental variables [16–18].

This study is grounded in the Transactional Model of Stress and Coping, which views stress as a dynamic interaction between environmental demands and an individual’s perceived coping resources [19]. According to this framework, when academic loads exceed coping capabilities, students may adopt emotion coping strategies to regulate psychological distress. From this perspective, emotional eating can be viewed as one coping behavior, whereby individuals consume food in response to negative emotional states rather than physiological hunger.

Within this framework, emotional eating has been widely recognized as a behavioral response to psychological stress and negative emotions [20]. Individuals experiencing stress may have difficulty distinguishing between physiological hunger and the desire to eat to alleviate emotional discomfort such as anxiety, irritability, or sadness [21, 22]. As a result, emotional eating is often associated with increased consumption of energy-dense and highly palatable foods, including sweets, snacks, sugary beverages, and fast foods, alongside reduced intake of healthier foods such as fruits and vegetables [23, 24]. While this behavior may provide temporary emotional relief, it may also contribute to unhealthy dietary patterns and adverse health outcomes over time [20, 25].

**Fig. 1:**
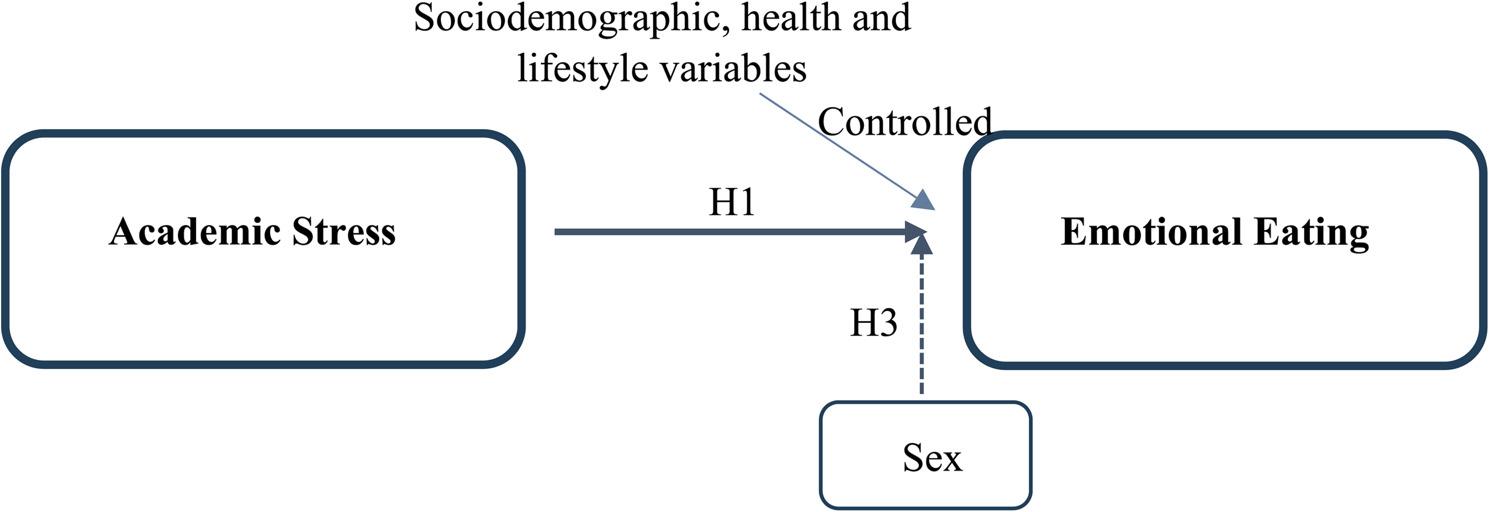
Conceptual framework of the study illustrating the hypothesized association between academic stress and emotional eating among postgraduate students. Academic stress represents the primary independent variable predicting emotional eating. Sex was examined as a potential moderator of the association between academic stress and emotional eating. Solid arrows indicate hypothesized direct associations, thin arrows refer to variables accounted for in the adjusted model, whereas dashed arrows represent the moderating effect of sex

Although previous studies have examined the relationship between academic stress and emotional eating among university students, most have focused on undergraduate populations or medical students [26–28]. Evidence regarding postgraduate students remains relatively limited despite the distinct academic pressures associated with graduate-level study. Furthermore, research examining this relationship among postgraduate students in Saudi Arabia remains limited. Understanding how academic stress influences emotional eating behavior among postgraduate students is crucial for developing targeted health promotion strategies within university environments. Therefore, the current study aims to examine the association between academic stress and emotional eating behavior among postgraduate students at King Abdulaziz University in Jeddah, Saudi Arabia. Based on the Transactional Model of Stress and Coping, the following hypotheses were developed (Fig. 1):

- H1: High levels of academic stress are associated with increased emotional eating.
- H2: The association between academic stress and emotional eating remains significant after adjusting for sociodemographic, health, and lifestyle variables.
- H3: Sex moderates the relationship between academic stress and emotional eating.

## Methods

### Study design

To examine the association between academic stress and emotional eating behavior, a cross-sectional study was conducted among postgraduate students at King Abdulaziz University in Jeddah, Saudi Arabia. An online, self-administered questionnaire was distributed during the study period from September 2024 to March 2025.

### Participants and sampling

Convenience sample of postgraduate students (male and female) from different postgraduate programs at King Abdulaziz University in Jeddah, Saudi Arabia, were eligible to participate in this study. Undergraduate students and students from other universities were excluded. Additionally, KAU does not offer part-time or distance-learning postgraduate programs; therefore, these were not applicable for this study.

The required sample size in this study was determined using the online Raosoft sample size calculator, with a confidence level of 95%, a margin of error of 5%, and a population size of around 12,000 postgraduate students, based on data from the Deanship of Admission and Registration at King Abdulaziz University [29, 30]. The estimated minimum required sample size was 373 students. The study was conducted in accordance with the Declaration of Helsinki and approved by the Unit of Biomedical Ethics Research Committee at King Abdulaziz University, Jeddah, number (Reference No. 270 − 24).

### Survey instrument

The online, self-administered questionnaire included four main sections (Supplementary 1).

#### Section 1

Sociodemographic, health, and lifestyle characteristics, including age, sex, nationality, marital status, academic level, faculty, monthly income, employment status, and residence status. Health and lifestyle characteristics were also evaluated, such as the existence of self-reported medical disorders, a self-reported history of depression, cigarette smoking, and physical activity, which was assessed using the frequency of moderate-intensity and vigorous-intensity exercise, with responses ranging from never to more than five times per week.

#### Section 2

Academic performance was measured via self-reported current Grade Point Average (GPA). Self-reported overall GPA has frequently been used as a measure of academic achievement in similar studies [31]. A student’s GPA was classified according to the grading system at King Abdulaziz University (KAU). Out of 5.0, a GPA of 3.75–3.99 is categorized as above average, 4.00–4.49 as very good, 4.50–4.47 as superior, and 4.75–5.0 as excellent [32].

#### Section 3

The validated Students’ Stress Inventory (SSI) was used to assess academic stress levels. The questionnaire captured perceived academic stress during the semester and was not limited to exam periods. The questionnaire and its scoring were adopted from a previously published source [33], which comprises four independent self-report subscales: (1) physical factor, (2) interpersonal relationship factor, (3) academic factor, and (4) environmental factor. The current study focused only on the third subscale (academic factor), which consists of 10 items, because it specifically measures stress related to academic demands such as coursework, deadlines, and academic performance expectations, which are related to the research aim. This approach is consistent with previous research that has used the academic factor subscale independently to examine academic stress [34]. The study has reported validity and reliability for the use of the academic subscale to measure academic stress [34]. Internal consistency in the current sample was assessed using Cronbach’s alpha. The SSI academic subscale (10 items) demonstrated good reliability (α = 0.867). Responses were recorded on a 4-point ordinal scale ranging from (Never “1,” Sometimes “2,” Often “3,” and Always “4”). Total scores range from 10 to 40, with higher scores indicating a higher level of academic stress. Specifically, scores are categorized as follows: a score of 10–18 indicates mild academic stress; 19–29 indicates moderate academic stress; and 30–40 indicates severe academic stress.

#### Section 4

Emotional eating behavior was assessed using the validated Emotional Eater Questionnaire (EEQ), which was adopted from a previously published source [35],which consists of 10 items; each has four response options (Never “0,” Sometimes “1,” Generally “2,” and Always “3”). Total scores range from 0 to 30 and were categorized as follows: scores of 0–5 were classified as a non-emotional eater; 6–10 were classified as a low emotional eater; 11–20 were classified as an emotional eater; and 21–30 were classified as a very emotional eater. Internal consistency in the current sample was assessed using Cronbach’s alpha. The EEQ (10 items) showed good reliability (α = 0.885).

To administer the questionnaire to participants in their native language, two research experts translated items from English into Arabic; these were then independently back-translated into English by a third researcher to ensure that the translation closely resembled the original survey and the accuracy of the translation. The survey was pilot tested through face and content validity. The questionnaire was reviewed and revised by three experts in the field of nutrition and seven female university students due to accessibility and availability during the pilot test, to ensure that all questions were well defined, clearly understood, and free from difficulties related to the content or language.

The online questionnaire was sent through the Deanship of Graduate Studies to all students via their official university email. The questionnaire was also distributed via social media platforms such as WhatsApp, Telegram, Snapchat, and X. To prevent duplicate responses, the survey platform restricted multiple submissions from the same device/IP address. Information regarding the study objectives was included on the first page of the questionnaire, and informed consent was obtained electronically from each participant before they began the questionnaire. Participants were required to indicate their consent by selecting an agreement option before starting the questionnaire.

### Statistical analysis

The data were analyzed using the Statistical Package for Social Sciences version 26.0 (SPSS Inc., Chicago, USA). Descriptive statistics, including frequencies and percentages, were used to describe the categorical and quantitative variables; mean and standard deviation were used to describe continuous variables.

The association between academic stress and emotional eating behavior was assessed by Pearson’s chi-square test and Spearman’s correlation coefficient. Pearson’s chi-square was used to examine associations between categorized academic stress levels and categorized emotional eating. Spearman’s correlation was used to assess the association between SSI total score and EEQ total score.

An ordinal logistic regression was fitted with emotional eating category as the ordinal outcome and academic stress level (mild, moderate, and severe) as the primary predictor. In addition to the main effects, a moderation analysis was conducted to examine whether sex moderates the association between academic stress and emotional eating. An interaction term between academic stress level and sex was added to the adjusted ordinal logistic regression model. Both the main-effects and moderation models were adjusted for potential confounders, including age, marital status, university level, study stage, employment status, income, smoking status, physical activity, depression diagnosis, and GPA; with sex included as both a main effect and an interaction term. The proportional odds assumption was tested and met. Model estimates are presented as β coefficients. Odds ratios (OR) and 95% confidence intervals (CI) were obtained by exponentiating the β coefficients and their corresponding confidence limits. A *P*-value of < 0.05 was considered statistically significant.

## Results

### Characteristics of study participants

A total of 505 participants were included in the study, and Table 1 presents their characteristics. The majority were between 23 and 30 years of age (63.4%), and 94.1% of participants were Saudis. Most (85.9%) were female, and more than half (54.9%) were unmarried. Roughly 55.8% of the participants reported having a monthly income below 4,000 SAR (1066.52 USD). More than half of the participants (67.3%) were full-time students, and the majority (79.8%) lived with their families. Participants were primarily master’s students (84.0%), and 59.4% were in the coursework stage. Approximately 66.7% reported a cumulative GPA between 4.75 and 5. More than half of the participants (53.3%) were from the Faculty of Humanities and Social Studies. Of the participants, 21.2% reported medical conditions, although 13.7% of the participants had been previously diagnosed with depression. In terms of physical activity, 56.5% reported rarely engaging in moderate or vigorous exercise, and 78.7% reported that they never did so. The majority (92.1%) were non-smokers.

**Table 1:** Characteristics of study participants

	*N*	%
Demographic characteristics (n = 505)		
Age		
23–26 yrs	163	32.3%
27–30 yrs	157	31.1%
30–34 yrs	99	19.6%
35 + yrs	86	17.0%
Nationality		
Saudi	475	94.1%
Non-Saudi	30	5.9%
Sex		
Female	434	85.9%
Male	71	14.1%
Marital Status		
Single	277	54.9%
Married	202	40.0%
Divorced	25	5.0%
Widowed	1	0.2%
Monthly income		
< 4,000 SAR	282	55.8%
4,000–5,999 SAR	41	8.1%
6,000 SAR − 7,999 SAR	43	8.5%
8,000–9,999 SAR	41	8.1%
10,000 + SAR	98	19.4%
Employment Status		
Student	340	67.3%
Student with part time employment	56	11.1%
Student with full time employment	109	21.6%
Housing Status		
Living alone (rented/owned)	57	11.3%
Living with family	403	79.8%
University accommodation (away from family)	45	8.9%
Academic characteristics		
University Stage (e.g., Bachelor, Master)		
Master’s	424	84.0%
PhD	81	16.0%
Academic Year Level		
Courses study stage	300	59.4%
Thesis work stage	205	40.6%
Faculty		
Human and Life Sciences	165	32.7%
Engineering and IT	40	7.9%
Medicine Medical Sciences	31	6.1%
Humanities and Social Studies	269	53.3%
Cumulative GPA		
3.75–3.99	19	3.8%
4–4.49	54	10.7%
4.50–4.74	95	18.8%
4.75–5	337	66.7%
Health and lifestyle characteristics		
Medical condition		
No	398	78.8%
Yes	107	21.2%
Diabetes	20	4.0%
Blood pressure	12	2.4%
Food allergy	3	0.6%
Other	80	15.8%
Previous depression diagnosis		
No	436	86.3%
Yes	69	13.7%
Frequency of Moderate Exercise (e.g., walking, swimming)		
Never	124	24.6%
Rarely	161	31.9%
1–2 times/week	127	25.1%
3–4 times/week	67	13.3%
More than 5 times/week	26	5.1%
Frequency of Vigorous Exercise (e.g., heavy lifting, fast cycling)		
Never	222	44.0%
Rarely	175	34.7%
1–2 times/week	66	13.1%
3–4 times/week	37	7.3%
More than 5 times/week	5	1.0%
Cigarette smoking		
No	465	92.1%
Yes	30	5.9%
Ex-smoker	10	2.0%

### Academic stress levels among participants

**Fig. 2:**
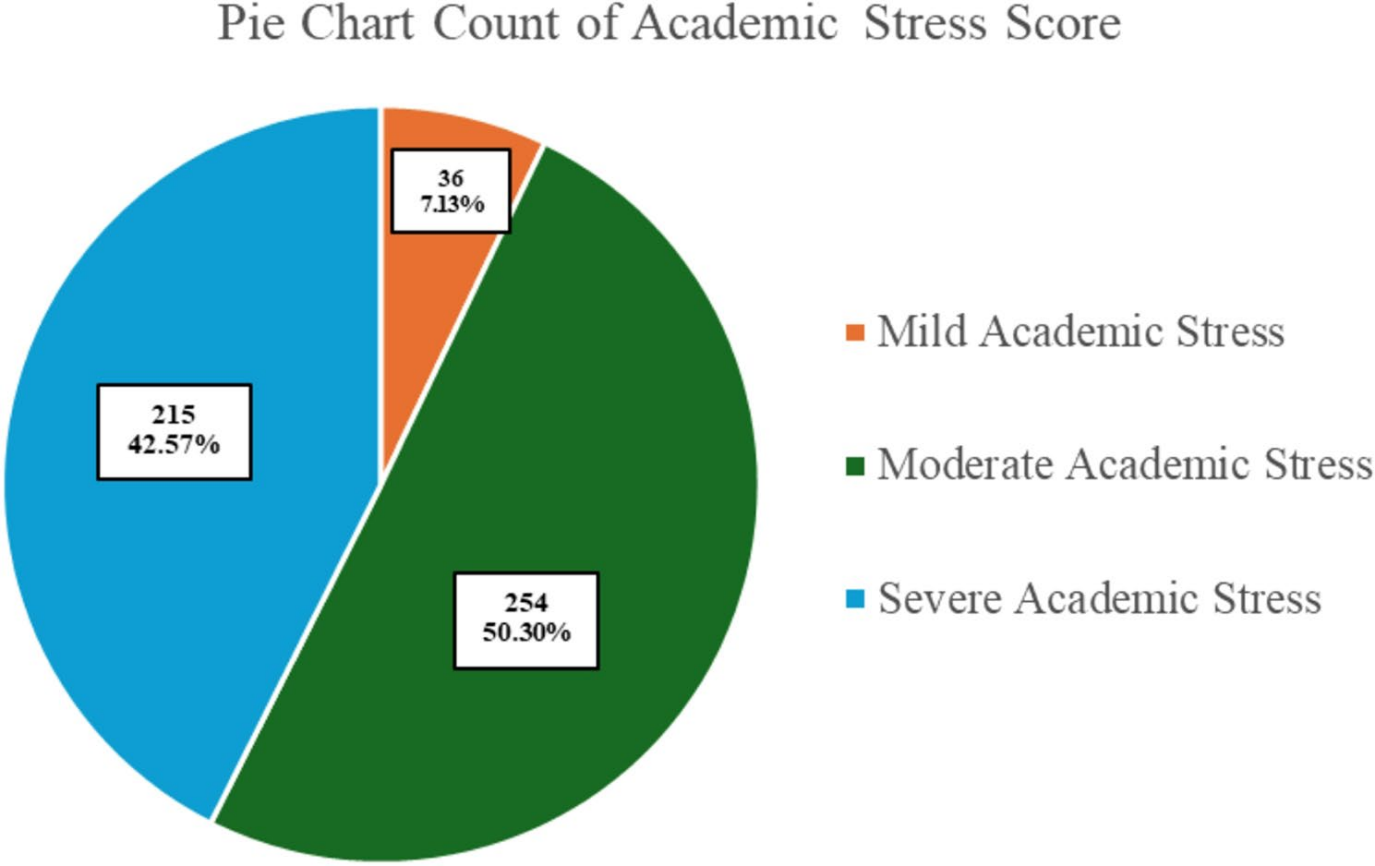
Distribution of academic stress among students

Within the study population, the total mean of academic stress scores was 27.59 ± 6.54, indicating moderate levels of academic stress. Half of the participants (50.3%) were categorized into this moderate level, followed by those who experienced severe academic stress (42.6%); only 7.1% were classified as experiencing mild academic stress (Fig. 2).

Table 2 summarizes the association between participant characteristics and academic stress levels. A significantly higher proportion of female students were classified as experiencing severe academic stress as compared with male students (45.2% vs. 26.8%, *P* < 0.001). Monthly income was also significantly associated with academic stress level (*P* = 0.007), such that higher academic stress was more prevalent among students with lower income (< 4,000 SAR, equivalent to < 1066.52 USD). Lower frequency of physical activity was also significantly associated with severe academic stress, as students who never engaged in moderate (*P* = 0.008) or vigorous (*P* = 0.007) exercise reported higher levels of severe stress. Other demographic variables, such as age, marital status, academic level, faculty, and GPA, were not significantly associated with academic stress levels.

**Table 2:** Characteristics of study participants by academic stress score

	Mild Academic Stress *n* = 36	Moderate Academic Stress *n* = 254	Severe Academic Stress *n* = 215	*P*-value
Demographic characteristics				
Age				
23–26 yrs	6 (3.7%)	76 (46.6%)	81 (49.7%)	0.096
27–30 yrs	14 (8.9%)	80 (51%)	63 (40.1%)	
30–34 yrs	6 (6.1%)	57 (57.6%)	36 (36.4%)	
35 + yrs	10 (11.6%)	41 (47.7%)	35 (40.7%)	
Nationality				
Saudi	34 (7.2%)	238 (50.1%)	203 (42.7%)	0.943
Non-Saudi	2 (6.7%)	16 (53.3%)	12 (40%)	
Sex				
Female	24 (5.5%)	214 (49.3%)	196 (45.2%)	< 0.001
Male	12 (16.9%)	40 (56.3%)	19 (26.8%)	
Marital Status				
Single	16 (5.8%)	142 (51.3%)	119 (43%)	0.675
Married	17 (8.4%)	99 (49%)	86 (42.6%)	
Divorced	3 (12%)	13 (52%)	9 (36%)	
Widowed	0 (0%)	0 (0%)	1 (100%)	
Monthly income				
< 4,000 SAR	12 (4.3%)	130 (46.1%)	140 (49.6%)	0.007
4,000–5,999 SAR	3 (7.3%)	22 (53.7%)	16 (39%)	
6,000 SAR − 7,999 SAR	4 (9.3%)	24 (55.8%)	15 (34.9%)	
8,000–9,999 SAR	3 (7.3%)	24 (58.5%)	14 (34.1%)	
10,000 + SAR	14 (14.3%)	54 (55.1%)	30 (30.6%)	
Employment Status				
Student	20 (5.9%)	170 (50%)	150 (44.1%)	0.335
Student with part time employment	4 (7.1%)	32 (57.1%)	20 (35.7%)	
Student with full time employment	12 (11%)	52 (47.7%)	45 (41.3%)	
Housing Status				
Living alone (rented/owned)	3 (5.3%)	29 (50.9%)	25 (43.9%)	0.607
Living with family	32 (7.9%)	203 (50.4%)	168 (41.7%)	
University accommodation (away from family)	1 (2.2%)	22 (48.9%)	22 (48.9%)	
Academic characteristics				
University Stage (e.g., Bachelor, Master)				
Master’s	27 (6.4%)	212 (50%)	185 (43.6%)	0.236
PhD	9 (11.1%)	42 (51.9%)	30 (37%)	
Academic Year Level				
Courses study stage	16 (5.3%)	148 (49.3%)	136 (45.3%)	0.091
Thesis work stage	20 (9.8%)	106 (51.7%)	79 (38.5%)	
Faculty				
Human and Life Sciences	12 (7.3%)	88 (53.3%)	65 (39.4%)	0.114
Engineering and IT	2 (5%)	16 (40%)	22 (55%)	
Medicine Medical Sciences	0 (0%)	12 (38.7%)	19 (61.3%)	
Humanities and Social Studies	22 (8.2%)	138 (51.3%)	109 (40.5%)	
Cumulative GPA				
3.75–3.99	0 (0%)	9 (47.4%)	10 (52.6%)	0.604
4–4.49	2 (3.7%)	26 (48.1%)	26 (48.1%)	
4.50–4.74	9 (9.5%)	50 (52.6%)	36 (37.9%)	
4.75–5	25 (7.4%)	169 (50.1%)	143 (42.4%)	
Health and lifestyle characteristics				
Medical condition				
No	31 (7.8%)	205 (51.5%)	162 (40.7%)	0.198
Yes	5 (4.7%)	49 (45.8%)	53 (49.5%)	
Diabetes	2 (10%)	8 (40%)	10 (50%)	0.622
Blood pressure	1 (8.3%)	5 (41.7%)	6 (50%)	0.721
Food allergy	0 (0%)	1 (33.3%)	2 (66.7%)	0.614
Other	4 (5%)	36 (45%)	40 (50%)	0.306
Previous depression diagnosis				
No	31 (7.1%)	225 (51.6%)	180 (41.3%)	0.311
Yes	5 (7.2%)	29 (42%)	35 (50.7%)	
Frequency of Moderate Exercise (e.g., walking, swimming)				
Never	3 (2.4%)	53 (42.7%)	68 (54.8%)	0.008
Rarely	8 (5%)	83 (51.6%)	70 (43.5%)	
1–2 times/week	15 (11.8%)	67 (52.8%)	45 (35.4%)	
3–4 times/week	8 (11.9%)	37 (55.2%)	22 (32.8%)	
More than 5 times/week	2 (7.7%)	14 (53.8%)	10 (38.5%)	
Frequency of Vigorous Exercise (e.g., heavy lifting, fast cycling)				
Never	7 (3.2%)	105 (47.3%)	110 (49.5%)	0.007
Rarely	13 (7.4%)	97 (55.4%)	65 (37.1%)	
1–2 times/week	11 (16.7%)	30 (45.5%)	25 (37.9%)	
3–4 times/week	5 (13.5%)	19 (51.4%)	13 (35.1%)	
More than 5 times/week	0 (0%)	3 (60%)	2 (40%)	
Cigarette smoking				
No	32 (6.9%)	234 (50.3%)	199 (42.8%)	0.889
Yes	3 (10%)	14 (46.7%)	13 (43.3%)	
Ex-smoker	1 (10%)	6 (60%)	3 (30%)	

The chi-square test was used to examine associations between categorized variables

### Emotional eating score among participants

**Fig. 3:**
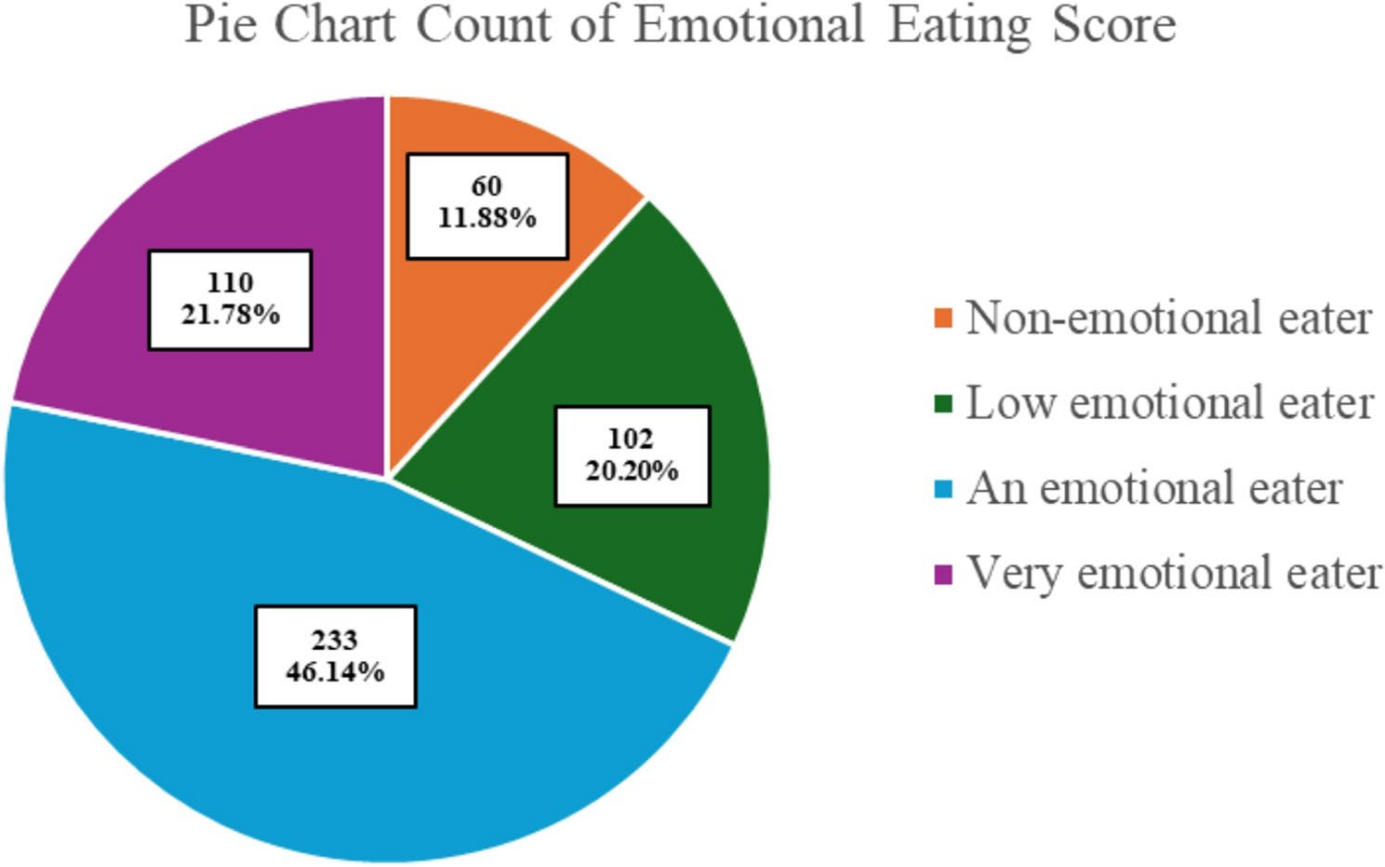
Emotional eating categories

Students’ mean emotional eating score was 14.42 ± 7.28, which indicated moderate emotional eating behavior. As Fig. 3 reflects, the majority of study participants (67.9%) were classified as either emotional or very emotional eaters. In terms of demographic variables, only sex was associated with emotional eating score, as 24% of female students were very emotional eaters, compared with just 8.5% of males (*P* = 0.031; see Table 3).

**Table 3:** Characteristics of study participants by emotional eating score

	Non-emotional eater *n* = 60	Low emotional eater *n* = 102	An emotional eater *n* = 233	Very emotional eater *n* = 110	*P*-value
Demographic characteristics					
Age					
23–26 yrs	16 (9.8%)	31 (19%)	71 (43.6%)	45 (27.6%)	0.222
27–30 yrs	19 (12.1%)	30 (19.1%)	80 (51%)	28 (17.8%)	
30–34 yrs	14 (14.1%)	27 (27.3%)	37 (37.4%)	21 (21.2%)	
35 + yrs	11 (12.8%)	14 (16.3%)	45 (52.3%)	16 (18.6%)	
Nationality					
Saudi	56 (11.8%)	94 (19.8%)	219 (46.1%)	106 (22.3%)	0.623
Non-Saudi	4 (13.3%)	8 (26.7%)	14 (46.7%)	4 (13.3%)	
Sex					
Female	49 (11.3%)	86 (19.8%)	195 (44.9%)	104 (24%)	**0.031**
Male	11 (15.5%)	16 (22.5%)	38 (53.5%)	6 (8.5%)	
Marital Status					
Single	36 (13%)	60 (21.7%)	125 (45.1%)	56 (20.2%)	0.782
Married	22 (10.9%)	37 (18.3%)	97 (48%)	46 (22.8%)	
Divorced	2 (8%)	5 (20%)	11 (44%)	7 (28%)	
Widowed	0 (0%)	0 (0%)	0 (0%)	1 (100%)	
Monthly income					
< 4,000 SAR	32 (11.3%)	58 (20.6%)	124 (44%)	68 (24.1%)	0.276
4,000–5,999 SAR	3 (7.3%)	7 (17.1%)	19 (46.3%)	12 (29.3%)	
6,000 SAR − 7,999 SAR	8 (18.6%)	7 (16.3%)	23 (53.5%)	5 (11.6%)	
8,000–9,999 SAR	8 (19.5%)	6 (14.6%)	17 (41.5%)	10 (24.4%)	
10,000 + SAR	9 (9.2%)	24 (24.5%)	50 (51%)	15 (15.3%)	
Employment Status					
Student	41 (12.1%)	69 (20.3%)	159 (46.8%)	71 (20.9%)	0.400
Student with part time employment	6 (10.7%)	16 (28.6%)	19 (33.9%)	15 (26.8%)	
Student with full time employment	13 (11.9%)	17 (15.6%)	55 (50.5%)	24 (22%)	
Housing Status					
Living alone (rented/owned)	4 (7%)	10 (17.5%)	32 (56.1%)	11 (19.3%)	0.326
Living with family	54 (13.4%)	82 (20.3%)	181 (44.9%)	86 (21.3%)	
University accommodation (away from family)	2 (4.4%)	10 (22.2%)	20 (44.4%)	13 (28.9%)	
Academic characteristics					
University Stage (e.g., Bachelor, Master)					
Master’s	47 (11.1%)	81 (19.1%)	200 (47.2%)	96 (22.6%)	0.214
PhD	13 (16%)	21 (25.9%)	33 (40.7%)	14 (17.3%)	
Academic Year Level					
Courses study stage	32 (10.7%)	64 (21.3%)	138 (46%)	66 (22%)	0.704
Thesis work stage	28 (13.7%)	38 (18.5%)	95 (46.3%)	44 (21.5%)	
Faculty					
Human and Life Sciences	23 (13.9%)	33 (20%)	80 (48.5%)	29 (17.6%)	0.655
Engineering and IT	3 (7.5%)	8 (20%)	16 (40%)	13 (32.5%)	
Medicine Medical Sciences	2 (6.5%)	5 (16.1%)	16 (51.6%)	8 (25.8%)	
Humanities and Social Studies	32 (11.9%)	56 (20.8%)	121 (45%)	60 (22.3%)	
Cumulative GPA					
3.75–3.99	2 (10.5%)	4 (21.1%)	10 (52.6%)	3 (15.8%)	0.963
4–4.49	7 (13%)	12 (22.2%)	23 (42.6%)	12 (22.2%)	
4.50–4.74	10 (10.5%)	24 (25.3%)	41 (43.2%)	20 (21.1%)	
4.75–5	41 (12.2%)	62 (18.4%)	159 (47.2%)	75 (22.3%)	
Health and lifestyle characteristics					
Medical condition					
No	50 (12.6%)	83 (20.9%)	179 (45%)	86 (21.6%)	0.634
Yes	10 (9.3%)	19 (17.8%)	54 (50.5%)	24 (22.4%)	
Diabetes	4 (20%)	2 (10%)	11 (55%)	3 (15%)	0.376
Blood pressure	2 (16.7%)	1 (8.3%)	6 (50%)	3 (25%)	0.705
Food allergy	0 (0%)	1 (33.3%)	1 (33.3%)	1 (33.3%)	0.751
Other	6 (7.5%)	16 (20%)	40 (50%)	18 (22.5%)	0.600
Previous depression diagnosis					
No	56 (12.8%)	93 (21.3%)	198 (45.4%)	89 (20.4%)	0.054
Yes	4 (5.8%)	9 (13%)	35 (50.7%)	21 (30.4%)	
Frequency of Moderate Exercise (e.g., walking, swimming)					
Never	12 (9.7%)	25 (20.2%)	51 (41.1%)	36 (29%)	0.111
Rarely	19 (11.8%)	34 (21.1%)	81 (50.3%)	27 (16.8%)	
1–2 times/week	16 (12.6%)	21 (16.5%)	59 (46.5%)	31 (24.4%)	
3–4 times/week	9 (13.4%)	20 (29.9%)	25 (37.3%)	13 (19.4%)	
More than 5 times/week	4 (15.4%)	2 (7.7%)	17 (65.4%)	3 (11.5%)	
Frequency of Vigorous Exercise (e.g., heavy lifting, fast cycling)					
Never	26 (11.7%)	46 (20.7%)	96 (43.2%)	54 (24.3%)	0.233
Rarely	19 (10.9%)	33 (18.9%)	88 (50.3%)	35 (20%)	
1–2 times/week	6 (9.1%)	14 (21.2%)	32 (48.5%)	14 (21.2%)	
3–4 times/week	8 (21.6%)	9 (24.3%)	14 (37.8%)	6 (16.2%)	
More than 5 times/week	1 (20%)	0 (0%)	3 (60%)	1 (20%)	
Cigarette smoking					
No	56 (12%)	95 (20.4%)	211 (45.4%)	103 (22.2%)	0.366
Yes	3 (10%)	4 (13.3%)	16 (53.3%)	7 (23.3%)	
Ex-smoker	1 (10%)	3 (30%)	6 (60%)	0 (0%)	

The chi-square test was used to examine associations between categorized variables

### Association between academic stress levels and emotional eating behavior

**Fig. 4:**
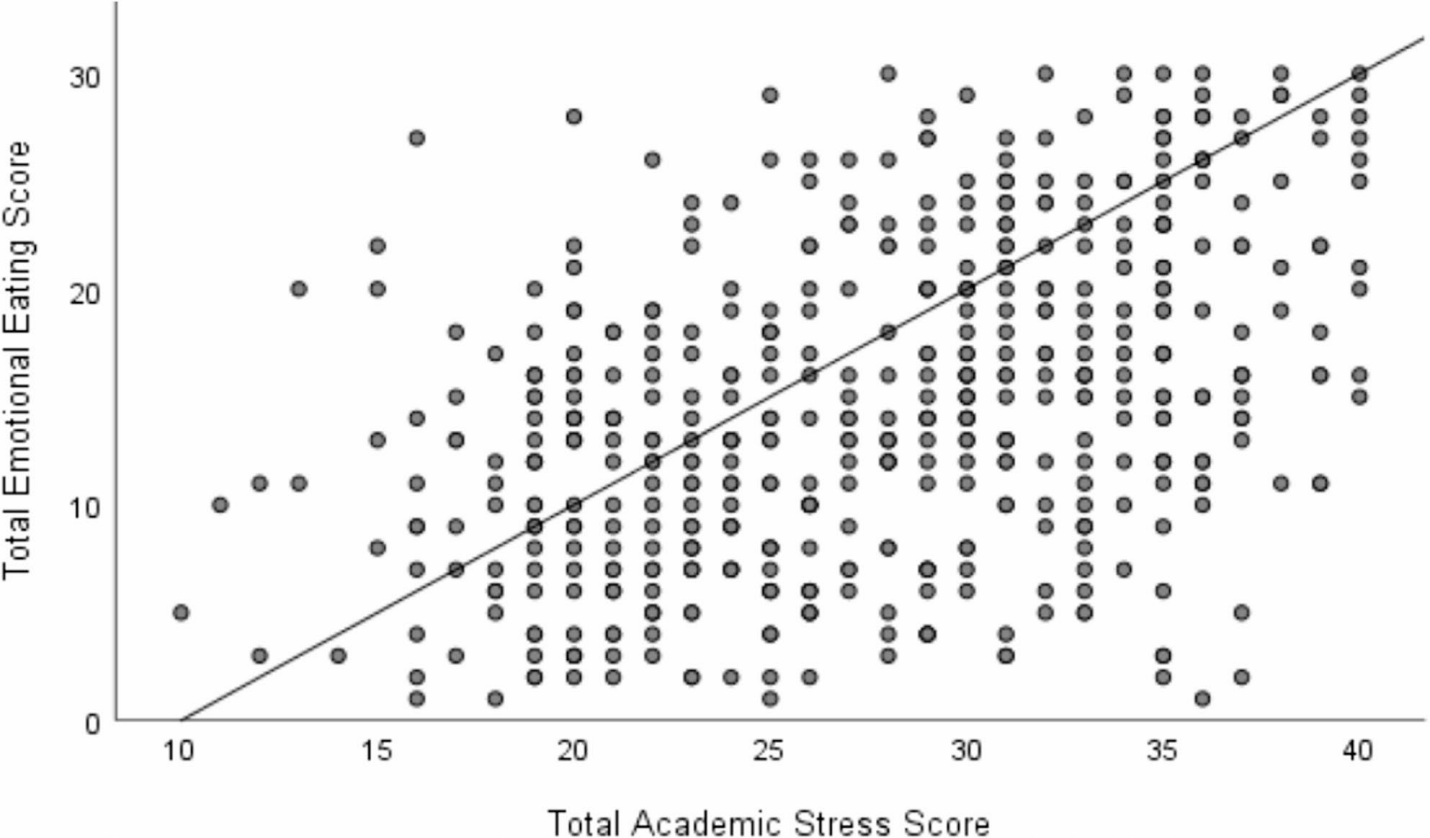
Correlation between academic stress and emotional eating scores. Spearman’s rank correlation was used to assess the association between SSI total score and EEQ total score

A statistically significant positive correlation was noted between academic stress and emotional eating scores (*r* = 0.429, *P* < 0.001; see Fig. 4), suggesting that higher academic stress is moderately correlated with greater emotional eating behavior.

The ordinal logistic regression analysis showed a statistically significant association between academic stress and emotional eating behavior (Table 4). Compared with students experiencing mild academic stress, those experiencing severe academic stress had significantly higher odds of being more severe emotional eaters (OR = 7.382, 95% CI [3.777, 14.43], *P* < 0.001). Students with moderate academic stress also showed increased odds of being more severe emotional eaters (OR = 1.738), but this association did not reach statistical significance (*P* = 0.090). The odds of the association between severe academic stress and severe emotional eating remained significant after adjusting for demographic, psychological, and lifestyle factors (adjusted OR = 6.633, 95% CI [3.294, 13.343], *P* < 0.001). Students with severe academic stress had more than six-fold higher odds of being classified as a more severe emotional eater, even after adjustment for demographic, academic, psychological, and lifestyle factors.

**Table 4:** Association between academic stress levels and emotional eating behavior (Ordinal logistic regression results)

Academic stress	Unadjusted OR (95% CI), *P*-value	Adjusted OR (95% CI), *P*-value
Mild	ref.	ref.
Moderate	1.738 (0.918–3.29), 0.09	1.675 (0.869–3.225), 0.123
Severe	7.382 (3.777–14.43), < 0.001***	6.633 (3.294–13.343), < 0.001***

Unadjusted ORs are derived from bivariate ordinal logistic regression. Adjusted ORs are derived from a multivariable model controlling for age, sex, marital status, university level, study stage, monthly income, employment status, physical activity (moderate and vigorous), depression diagnosis, smoking status, and GPA. The proportional odds assumption was tested and met. ORs and 95% CIs were obtained by exponentiating the regression coefficients and their confidence limits. *** indicates significance, *P* < 0.001

### Moderation analysis of sex in the association between academic stress and emotional eating behavior

An additional moderation analysis was conducted to test whether sex modified the association between academic stress and emotional eating (Table 5). Neither the interaction between sex and moderate academic stress nor the interaction between sex and severe academic stress was statistically significant (*P* = 0.842 and *P* = 0.830, respectively), indicating that sex did not moderate this association. Also, academic stage (*P* = 0.781) and university level (*P* = 0.182) were not significantly associated with emotional eating in the adjusted model.

**Table 5:** Moderation analysis of sex in the association between academic stress and emotional eating behavior

Variable	Adjusted OR (95% CI),	*P*-value
Sex (male vs. female)	0.683 (0.188–2.479)	0.562
Sex × moderate academic stress	1.157 (0.276–4.860)	0.842
Sex × severe academic stress	0.841 (0.173–4.088)	0.830
Academic stage (thesis vs. coursework)	1.051 (0.740–1.493)	0.781
University level (PhD vs. Master’s)	0.699 (0.413–1.183)	0.182

Results are from an adjusted ordinal logistic regression model including sex, academic stress, sex × academic stress interaction terms, academic stage, university level, age, marital status, monthly income, employment status, moderate and vigorous physical activity, previous depression diagnosis, smoking status, and GPA. The proportional odds assumption was tested and met

## Discussion

This study aimed to examine the association between academic stress and emotional eating among postgraduate students. Most participants reported moderate levels of academic stress and emotional eating. Academic stress was significantly associated with several sociodemographic and lifestyle factors, including sex, income level, and physical activity. A significant positive association was also observed between academic stress and emotional eating behavior, indicating that higher levels of academic stress were linked to greater emotional eating among postgraduate students. Female students reported higher levels of emotional eating compared with male students. However, the moderation analysis indicated that sex did not significantly modify the association between academic stress and emotional eating. These findings highlight the potential role of academic stress as an important factor associated with emotional eating among postgraduate students.

Academic stress is widely reported among postgraduate students across various educational settings. An Indian study found that the majority of postgraduate students (84.8%) experienced moderate academic stress, primarily due to examination-related pressures [36], which shows a higher prevalence than that observed in the present study. Similarly, research at Jimma University in Ethiopia reported high levels of academic stress among postgraduate students, with academic demands identified as the primary source of stress [37]. In the Saudi context, a study at King Khalid University found that students experienced considerable academic stress, with the most common stressors related to interactions with classmates, relationships with instructors, and the complexity of academic materials [38]. These findings are consistent with the present study, indicating that postgraduate students across different educational backgrounds experience substantial academic stress due to demanding academic expectations and interpersonal academic environments. Academic demands such as examinations, research responsibilities, tight schedules, and heavy coursework, as well as fear of failure and high personal or family expectations, have been frequently identified as key contributors to academic stress [10]. Such stress may negatively affect students’ psychological well-being and academic performance [15, 39]. Therefore, it is important to identify both internal and external factors that contribute to academic stress and to develop effective strategies that support students in managing these pressures and maintaining their academic success [40].

In the current study, sex was associated with academic stress level, such that female students were more likely to experience severe academic stress compared with male students. Our findings align with Swedish and Belgrade studies that reported higher levels of academic stress among female students as compared with males (Swedish: 16% of females vs. 3% of males; Belgrade: 28% of females vs. 19% of males) [16]. Among university students in Abha, Saudi Arabia, the trend in prevalence was the same, with approximately 76% of female students facing high academic stress, as compared with 58% of male students [8]. This suggests that females may be more sensitive to stressors and more likely to both express and report their feelings when they face stressful situations, in addition to demonstrating stronger perfectionistic tendencies, higher personal standards, higher perceived workload, and a greater likelihood of experiencing role overload, and this may be due to hormones or some other aspect of the nature of females [18, 41, 42].

This study indicated that academic stress was also significantly associated with lower income levels. Similar results were reported in studies conducted in Iraq and Ethiopia, where students with low monthly income experienced higher academic stress than those with high monthly income [43]. Insufficient income and financial constraints can cause students stress, impacting their lives in general and their academic lives in particular. Students need to ensure they can afford essential resources such as food, transportation, housing, academic materials, scientific activities, and other necessities. The lack of these resources is a source of stress and negatively affects their academic performance [43]. Furthermore, insufficient income can limit students’ social life and their access to sports and recreational activities. Given that such activities may relieve stress, they may also lead to better academic performance and quality of life [44]. Such findings agree with the findings in this study of a positive association between academic stress and low levels of physical activity. Studies from Puerto Rico and Sweden have reported that students who experienced higher levels of academic stress also had lower levels of physical activity [41, 45]. According to Cruz et al. (2013), students often engaged in physical activity as a way to cope with stress [45]. Physical activity helps in planning appropriate responses, managing emotions, completing tasks, and maintaining appropriate behavior, as it increases oxygenation and blood flow, which may explain how it eases stress [46–49]. Academic stress is a significant problem for university students, as the changes they experience during their studies tend to increase their exposure to stress. Therefore, it is suggested that students engage in physical activity as an effective way to manage academic stress [45].

Consistent with the Transactional Model of Stress and Coping, the findings suggest that academic stress, when evaluated as exceeding coping capacity, may trigger emotional coping behaviors such as emotional eating. Research has found that students are more likely to exhibit emotional eating behaviors during stressful study periods [50, 51]. During stressful situations, the sympathetic nervous system is activated, leading to increased secretion of cortisol and elevated blood glucose levels. As a result, individuals may experience an increased desire to consume food—especially calorie-dense foods—as a way to satisfy the body’s hormonal demand for glucose and temporarily alleviate negative emotions [52]. In this context, food consumption may serve as a short-term mechanism for mood regulation rather than a response to physiological hunger. A Malaysian study emphasized that students under academic stress often consumed unhealthy snacks as a form of emotional eating [24]. Other studies have shown that students with higher academic stress tend to increase their intake of sweets, sugary drinks, and fast food, while reducing their consumption of fruits and vegetables [23]. Long-term overconsumption of these foods can lead to weight gain, increased body mass index, and several health problems, such as inflammation, oxidative stress, and obesity [53]. Although causal inference is not possible in this cross-sectional design, the magnitude of the association indicates that severe academic stress is associated with relevant differences in emotional eating severity.

University students are at risk of obesity due to unhealthy eating behaviors and a lack of physical activity [54, 55]. Previous studies have indicated that physical activity can influence students’ eating behaviors, including emotional eating [56, 57], helping them to mitigate behaviors by improving their appetite control, preventing overeating, and reducing negative emotions [58–60]. Consistent with these findings, previous studies have found that 47%–54% of students are emotional eaters [50, 51, 61].

Despite the higher prevalence of emotional eating among female students, sex did not significantly moderate the relationship between academic stress and emotional eating in the current study, suggesting the association between academic stress and emotional eating remained similar across sexes. This aligns with previous research conducted among university students in Bahrain and Malaysia, where sex differences in emotional eating were observed [51, 62]. Several mechanisms may explain the higher levels of emotional eating reported among female students. This may be because female students may experience greater difficulty controlling their eating behaviors under stress [51]. Additionally, biological and psychosocial factors may contribute to these differences. Hormonal fluctuations associated with the menstrual cycle have been suggested to influence appetite and food cravings [41, 42]. A study conducted among university students in Boca Raton, Florida, showed that female students are more likely to use emotion-focused coping strategies, such as overeating, compared with males [63]. Furthermore, under stressful situations, female students may also show a greater tendency to select highly palatable, reward-oriented foods, which may further contribute to emotional eating behaviors [64]. This finding may indicate that academic stress acts as a common stressor influencing emotional eating behaviors regardless of gender.

Emotional eating is a coping strategy that often involves the overconsumption of foods rich in sugars and fats to escape negative or unpleasant feelings such as anger, sadness, fear, stress, and depression. Research indicates that these foods are known as “comfort foods” because they help alleviate unpleasant feelings and provide short-term pleasure [65], by releasing neurotransmitters such as dopamine and opiates in the brain, which boosts mood [53, 62]. Moreover, these palatable and energy-dense foods are commonly available on campus and are used as meal replacements, offering a quick energy boost during high-stress periods [66]. However, although emotional eating and consuming these foods may offer temporary comfort, in the long run, they can lead to negative outcomes such as obesity, weight gain, increased emotional distress, and feelings of guilt [67].

This study, which is the first in Saudi Arabia to examine the association between academic stress and emotional eating behavior among postgraduate students from various faculties at King Abdulaziz University in Jeddah, has several limitations. First, the study design cannot determine cause-and-effect. Furthermore, the findings should be interpreted with caution, as the study employed a non-probability convenience sampling method through an online survey, which may have introduced selection bias and response bias, as participation was voluntary and limited to students who had access to and chose to respond to the survey. The prevalence of female participants (85.9%) and the overrepresentation of certain faculties may limit the generalizability of the findings, particularly to male postgraduate students and to the broader postgraduate population. In addition, using a self-administered tool may limit precision and accuracy and introduce recall bias. Emotional eating was measured subjectively, rather than via dietary records, which would have potentially provided insights into the quality and quantity of the food at the time of consumption and factors affecting it. The academic stress was measured using a subscale of the SSI, as it specifically captures stressors related to university academic demands. However, this subscale has been previously validated and demonstrated acceptable psychometric properties in student populations.

## Practical implication

Despite these limitations, this study provides important findings about the correlation between academic stress and emotional eating behavior among the postgraduate students, an important topic for which data are limited in Saudi Arabia. The findings may help universities implement academic stress awareness campaigns and psychological support services, which may further contribute to preventing stress-related emotional eating behaviors. In addition, universities may incorporate regular physical activity services to improve emotional regulation and reduce unhealthy eating behaviors among postgraduate students.

## Conclusions

This study demonstrated a significant association between academic stress and emotional eating among postgraduate students in Saudi Arabia. Most of the study’s participants experienced moderate academic stress and have been found to engage in emotional eating behaviors. This study also revealed sex differences, with female students experiencing higher levels of both academic stress and emotional eating as compared with males; however, sex did not significantly modify the association between academic stress and emotional eating. Additionally, academic stress was associated with socioeconomic and lifestyle factors, including lower income and reduced physical activity.

These findings highlight the importance of integrating stress management and healthy coping strategies into university health promotion programs for postgraduate students. Universities may consider implementing structured stress management initiatives and promoting physical activity as part of student wellness policies to reduce reliance on unhealthy coping behaviors such as emotional eating.

Future research should further investigate the mechanisms underlying stress-related eating behaviors and evaluate the effectiveness of targeted interventions aimed at reducing emotional eating among postgraduate students. Qualitative studies may also provide deeper insights into students’ experiences with emotional eating and the types of foods consumed during periods of academic stress.

## Supplementary Information

Supplementary Material 1.

## Data Availability

The authors acknowledged that the research data are available upon request.
