# Supplementary material for: Academic stress is associated with emotional eating behavior among postgraduate students
Source: BMC Public Health. 2026 Apr 1;26:1530. doi: 10.1186/s12889-026-27233-3 (PMC13169801; doi:10.1186/s12889-026-27233-3)
Supplement: Supplementary file 1 — Supplementary Material 1. [file 12889_2026_27233_MOESM1_ESM.pdf]

Social and Demographic Characteristics

1. **Age**
   - 23 – 26 years
   - 27 – 30 years
   - 31 – 34 years
   - 35 years and above

2. **Nationality**
   - Saudi
   - Non-Saudi

3. **Gender**
   - Male
   - Female

4. **Marital Status**
   - Single
   - Married
   - Widowed
   - Divorced

5. **Academic Level:**
   - Master’s
   - PhD

6. **Academic Year Level:**
   - Courses study stage
   - Thesis work stage

7. **College:**
   - Medicine
   - Dentistry
   - Pharmacy
   - Applied Medical Science
   - Medical Rehabilitation Science
   - Nursing
   - Arts and Humanities
   - Economics and Administration
   - Human Science and Design
   - Education
   - Law
   - Institute of Arabic Language for Non-Native Speakers
   - Communication and Media
   - English Language Institute

- Engineering
- Computing and Information Technology
- Architecture and Planning
- Science
- Earth Science
- Marine Science
- Environmental Science
- Institute of Islamic Economics
- Maritime Studies

8. Monthly Income

- Less than 4,000 SAR
- 4,000 SAR to less than 6,000 SAR
- 6,000 SAR to less than 8,000 SAR
- 8,000 SAR to less than 10,000 SAR
- 10,000 SAR and above

9. Employment Status

- Student (Not affiliated with any employer)
- Student with part time employment
- Student with full time employment

10. Housing Status

- Living with family
- University accommodation (away from family)
- Living alone (rented/owned)

11. How often do you do moderate-intensity exercises such as walking, swimming, or running?

- Never
- Rarely
- 1-2 times/week
- 3-4 times/week
- More than 5 times/week

12. How often do you do vigorous exercise that causes sweating or difficulty breathing, such as lifting heavy objects, aerobics, or fast cycling?

- Never
- Rarely
- 1 – 2 times/week
- 3 – 4 times/week
- More than 5 times/week

13. Have you been diagnosed with any of the following (may chose all that is applicable)?

- I have not been diagnosed

- Diabetes
- Blood pressure
- Food allergies
- Other

14. If you answered “Other” to the previous question, please specify the condition/disease:

- …………………………………….

15. Have you been diagnosed with depression?

- Yes
- No

16. Do you smoke cigarettes?

- Yes, I smoke
- No, I do not smoke
- Ex-smoker

Cumulative GPA

1. What is your cumulative GPA?

- 4.75 – 5
- 4.50 – 4.74
- 4 – 4.49
- 3.75 – 3.99

Academic Stress

1: I have a financial problem because of the expenses of the university

- Never
- Somewhat frequent
- Frequent
- Always

2: I find difficult to juggle time between study and social activity

- Never
- Somewhat frequent
- Frequent
- Always

3: I feel nervous delivering the class presentation

- Never
- Somewhat frequent
- Frequent
- Always

4: I feel stressed as submission deadline neared

- Never
- Somewhat frequent
- Frequent
- Always

5: I feel stressed to sit for examination

- Never
- Somewhat frequent
- Frequent
- Always

6: I find difficult to juggle time between study and society involvement

- Never
- Somewhat frequent
- Frequent
- Always

7: I loss interest towards courses

- Never
- Somewhat frequent
- Frequent
- Always

8: I feel burden of academic workloads

- Never
- Somewhat frequent

- Frequent
- Always

9: I feel stressed dealing with difficult subject

- Never
- Somewhat frequent
- Frequent
- Always

10: I feel difficult in handling my academic problem

- Never
- Somewhat frequent
- Frequent
- Always

Emotional Eating Behavior

1: Do the weight scales have a great power over you? Can they change your mood?

- Never
- Sometimes
- Generally
- Always

2: Do you crave specific foods?

- Never
- Sometimes
- Generally
- Always

3: Is it difficult for you to stop eating sweet things, especially chocolate?

- Never
- Sometimes
- Generally
- Always

4: Do you have problems controlling the amount of certain types of food you eat?

- Never
- Sometimes
- Generally
- Always

5: Do you eat when you are stressed, angry or bored?

- Never
- Sometimes
- Generally
- Always

6: Do you eat more of your favourite food and with less control when you are alone?

- Never
- Sometimes
- Generally
- Always

7: Do you feel guilty when eat "forbidden" foods, like sweets or snacks?

- Never
- Sometimes
- Generally
- Always

8: Do you feel less control over your diet when you are tired after work at night?

- Never
- Sometimes
- Generally
- Always

9: When you overeat while on a diet, do you give up and start eating without control, particularly food that you think is fattening?

- Never
- Sometimes
- Generally
- Always

10: How often do you feel that food controls you, rather than you controlling food?

- Never
- Sometimes
- Generally
- Always
